# Adjuvant effect of oral Silymarin on patients’ wound healing process caused by thermal injuries

**DOI:** 10.22088/cjim.9.4.341

**Published:** 2018

**Authors:** Mohsen Mahmoodi-Nesheli, Shaabanali Alizadeh, Hassan Solhi, Jila Mohseni, Masomeh Mahmoodi-Nesheli

**Affiliations:** 1 Department of General Surgery, Medical school, Arak University of Medical Sciences, Arak, Iran; 2 Department of toxicology, Medical school, Arak University of Medical Sciences, Arak, Iran; 3 Department of Pharmacology, Medical school, Arak University of Medical Sciences, Arak, Iran; 4 Iran University of Medical Sciences, Tehran, Iran

**Keywords:** Thermal injury, Placebo effect, Silymarin, Wound Healing

## Abstract

**Background:**

According to the side effects of the usual treatment of burns, in improving the patients’ prognosis, there is a need to introduce faster and more effective adjuvant therapies to treat wounds, thus to improve the prognosis of patients. The aim of this study was to investigate the effect of adjuvant treatment with oral silymarin on wound healing process caused by second degree-burns among the patients admitted to Valiasr Hospital of Arak.

**Methods:**

This randomized, double-blind clinical trial study was carried out on 80 patients with second-degree burns covering 20 to 30 percent of the body with flame. Patients were randomized into 2 groups with an equal number: The intervention group (treated daily with oral 140-mg silymarin) and control (under the administration of placebo). Patients were treated for 8 weeks and at the end of weeks 1, 2, 3 and 4 according to the degree of wound healing (grade 1, 2 and 3) were followed during 2016-2017.

**Results:**

Changes in degrees of burn wound healing in both the intervention and placebo groups during the 4 weeks of treatment, significantly improved grade 3 completely. Based on the results, the complete remission in all four follow-up stages was significantly higher in silymarin group than the placebo group: Week 1 (intervention: 9 (22.5%), control: 0 (0%), (p=0.011), week 2 (intervention group: 18 (45%), placebo: 7 (17.5%), (p=0.000), week 3 (intervention: 24 (60%), placebo: 11 ( 27.5%), (p=0.051); week 4 (intervention: 27 (67.5%), control: 19 (47.5%), (p=0.003).

**Conclusions:**

According to our results, the 4-week adjuvant treatment with oral silymarin resulted in the full and faster wound recovery in patients with second degree-burn. So, it is recommended to use adjuvant treatments to obtain effective results.

Burns are the most extreme stress which can happen to the human body. The burn wounds can be the result of fires, electrical current, chemicals, boiling fluids, etc. (1). A significant number of burn cases are fire-related deaths. The actual percentage of burn is not specified at different levels and in different groups of the general population in the world, and it seems epidemiology of burns in different countries is influenced by various factors and has a different frequency, for example, it was found that the frequency of burns in India is higher among young women and in Europe, it is higher among middle-aged men than other groups in the general population (2). According to reports, it was found that the frequency of flame burns is approximately 3 to 10 percent and the average burn surface area in adults is approximately 20 % (1).

Now, three local compositions, including silver sulfadiazine, mafenide acetate, and silver nitrate are widely used to treat burns, but some adverse clinical side effects have been observed. So, due to the almost high prevalence of burns and to improve the prognosis of patients, it is recommended to introduce faster and more effective adjuvant therapies to treat burn wounds (1-3). According to antioxidant properties and its proper use in wound healing, it seems one of the useful drugs in healing burn wound is silymarin (silybum marianum extract) (4).

Silymarin is the most important active ingredient of silybum marianum or milk thistle which is composed of a group of chemicals called Flavonolignan (5). Silymarin has considerable antioxidant property and reduces free radicals inhibiting lipid peroxidation (SOD), and increases superoxide dismutase activity in the erythrocytes. In addition, it has anti-inflammatory effects and causes resistance to glutathione depletion and increases protein synthesis by hepatocytes during liver parenchymal injuries (6, 7). 

In several studies, the efficacy of silymarin in the treatment of chronic alcoholic liver disease, cirrhosis, fatty liver and viral hepatitis was indicated by assessing the metabolic, clinical and histological markers (1, 5); also the efficacy of silymarin has been introduced in reducing cholesterol and blood sugar levels, anticancer and immune regulatory effects (8, 9). In addition to the above, some authors have indicated in their animal studies that silymarin can be significantly effective in repairing skin wounds due to its antioxidant and anti-inflammatory properties (10, 11).

Although the different properties of silymarin have been examined for its effects on various disorders, very limited studies have been conducted on the effect of this drug in treating burn wounds. Given the need to introduce adjunctive therapy in patients with burns to improve the prognosis of these patients and consider the anti-inflammatory and antioxidant properties of silymarin, this study aimed to investigate the effect of adjuvant treatment with oral silymarin on wound healing process caused by second degree-burns among the patients admitted to Valiasr Hospital of Arak.

## Methods

In this clinical trial study followed during 2016-2017, 80 patients aged 18 to 45 years old with second-degree burns on 20 to 30 percent body surface area due to fire burn, referred to the emergency and burn unit of Valiasr Hospital of Arak participated after obtaining written consent and according to inclusion and exclusion criteria. Exclusion criteria were: electrical burns, chemical burns, children and infants, the presence of any chronic underlying and systemic disease (such as cardiovascular disease, metabolic diseases, immunosuppressed (diabetes, transplant), malignancy, inflammatory and infectious diseases, etc.), burns and trauma, pregnancy, drug addiction, respiratory tract burns, patients needing to intubation, patients with a GCS less than 15, recent history of silymarin consumption or a previous allergy and intolerance to oral silymarin, a history of malnutrition (malabsorption syndromes for the importance of wound healing). Classification of burn was diagnosed based on wound depth (5). 

After receiving basic clinical and demographic information (age, gender), patients were randomly (chosen by tossing the coin) divided into two groups of 40 samples. Patients in the intervention group received basic treatment and silymarin pill each day; in the control group, basic treatment and placebo were administered (with the same amount and shape of silymarin tablets produced by Goldaroo Company). Initial actions (ABCDE burn patterns, hydration, etc.) in dealing with burn patients and basic treatment with silver sulfadiazine, silver nitrate, and mafenide acetate were performed based on the condition of each patient. 

Wound healing in patients was evaluated at the end of weeks 1, 2, 3 and 4 based on clinical responses, for the first level: serious wounds, fresh and swollen; second level: granulation tissue on the wound; and third level: complete epithelial layer on the wounds (healing). At baseline and at the end of weeks 1, 2, 3 and 4, the pain was evaluated according to the visual analog scale, in which zero represents no pain and 10 indicates the unbearable pain (10). It should be noted that for as long as the patients are hospitalized, the administration and clinical examination was conducted by the hospital. 

Executive Assistant when patients were discharged, the prescribed drugs were given to patients, and the follow-up was performed by calling and visiting the patients back in the hospital. 

The data obtained from the examination form and demographic data were analyzed using SPSS Version 19 (SPSS Inc., Chicago, IL). The clinical trial ID registration of this research is IRCT2015070923138N1.

## Results

In this study 142 patients were enrolled, but after the initial examinations, 80 patients remained in two groups with 40 samples. Among 62 cases excluded from the study, 44 (70.9%) were not satisfied to enroll in the research and 18 (29.1%) cases had chronic systemic diseases.

**Figure 1:**
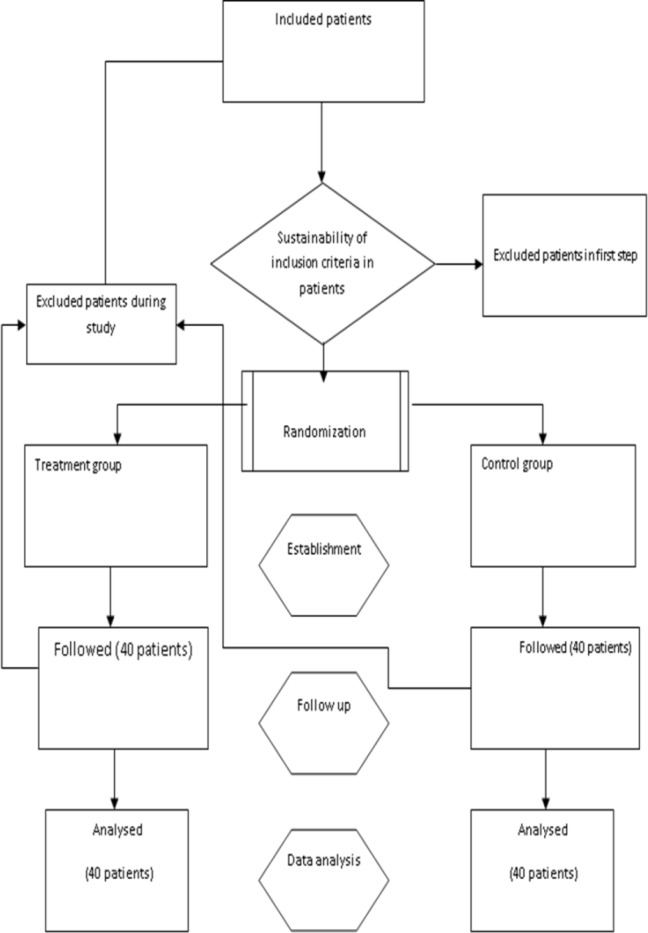
The flowchart of study, inclusion and exclusion of patients

During the study, 26 patients (10 patients in the placebo group and 16 patients in the intervention group) were also excluded from the study. Among these 26 cases, 12 (46.1%) subjects did not consent to continue to participate in the study and were not followed-up, 4 (15.4%) got a wound infection, and 10 (38.5%) due to lack of regular drug consumption were excluded (figure1)**.**

The average age of all patients was 33.1±5.6 years. The average age of the intervention group and placebo was 33.4±2.1 and 32.8±4.3 years, respectively, which had no significant difference (p=0.311). The frequency of male and female samples in the two groups were 43 (53.75%) and 37 (46.25%), respectively. So, there were 22 (55%) males and 18 (45%) females in the intervention group, and 21 (52.5%) males and 19 (47.5%) females in the placebo group. Gender distribution of patients was similar in both groups (p=0.2). Within 4 weeks and 3 levels of wound healing examinations, the changes in silymarin was significant in the first level (p=0.000), the second level (p=0.03) and the third level (p=0.001). 

**Figure 2:**
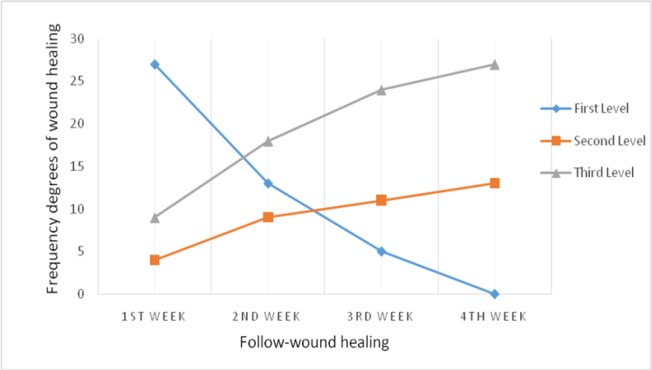
The frequency of changes during 4 weeks of wound healing in silymarin group

Hence, the frequency of the first and the second levels of wound healing during 4 weeks significantly reduced and the frequency of third level (healing) significantly increased (table 1). Changes in the level of wound healing during the 4 weeks of treatment in the silymarin group are shown in figure 2.

**Table 1:** The frequency of different levels of wound healing in patients of silymarin group within 4 weeks follow-up stages

**Time** **follow-up level **	**1** ^st^ ** week** **(40)**	**2** ^nd^ ** week** **(40)**	**3** ^rd^ ** week** **(40)**	**4** ^th^ ** week** **(40)**	**P-value** **(Friedman test)**
First * (%)	(67.5)27	(32.5)13	(12.5)5	(0)0	0.000
Second** (%)	(10)4	(22.5)9	(27.5)11	(32.5)13	0.03
Third*** (%)	(22.5)9	(45)18	(60)24	(67.5)27	0.001

* Serious wounds, fresh and swollen;

** granulation tissue on the wound;

*** complete epithelial layer on the wounds

**Figure 3:**
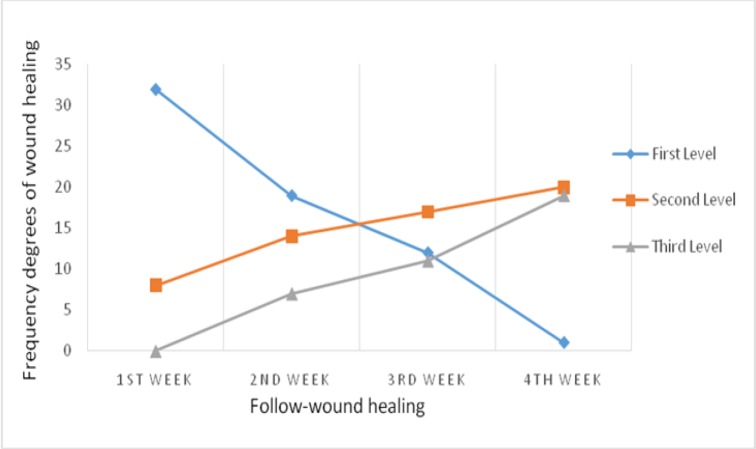
The frequency of changes during 4 weeks of wound healing in the placebo group

In the placebo group, changes in the frequency of first level (P=0.005), second level (P=0.041) and third level (P=0.021) of wound healing were significant. Consequently, the frequency of the first and the second levels of wound healing during 4 weeks significantly reduced and the frequency of the third level (healing) significantly increased. Changes in the level of wound healing during 4 weeks of treatment in the placebo group are shown in figure 3.

**Figure 4:**
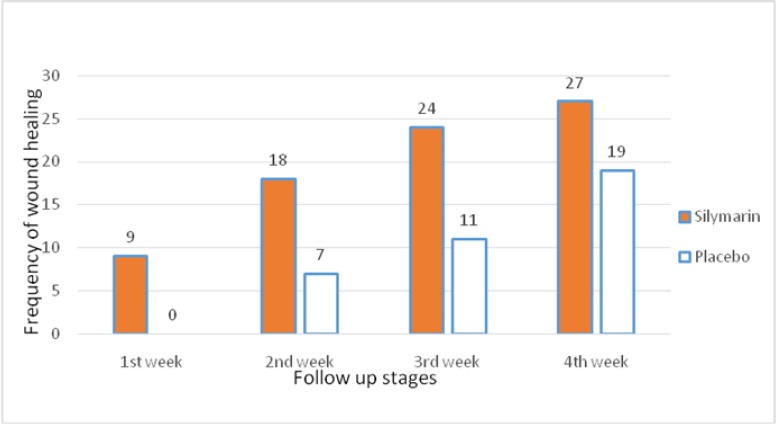
The comparison of the four follow-up stages of wound healing in burn patients in both groups of placebo and silymarin

Figure 4 shows the cases of complete remission (third level) between the intervention and placebo groups. Based on the results, the complete remission in all four follow-up stages was significantly higher in silymarin group than the placebo group: Week 1 (intervention group: 9 (22.5%), control: 0 (0%), (P=0.011), week 2 (intervention group: 18 (45%), placebo: 7 (17.5%), (P=0.000), week 3 (intervention: 24 (60%), placebo: 11 (27.5%), (P=0.051); week 4 (intervention: 27 (67.5%), control: 19 (47.5%), (P=0.003).

**Figure 5:**
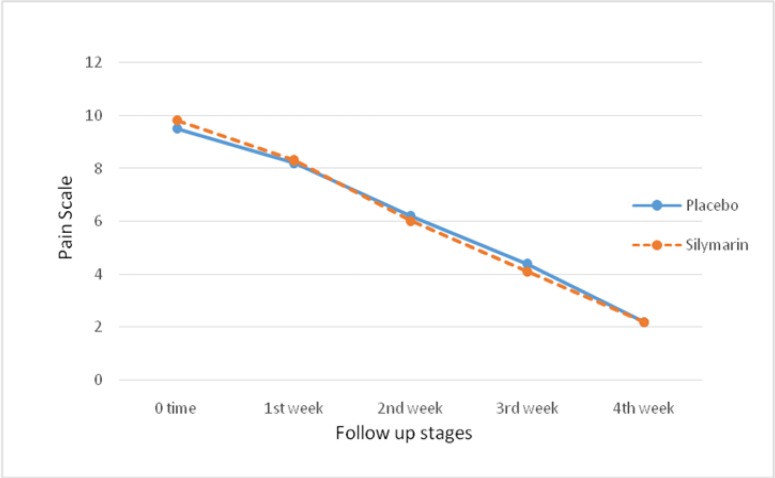
Changes in the mean visual analog pain scale during the 4 weeks of treatment of placebo and silymarin groups

The information about the mean visual analog pain scale in 4 follow-up stages and its comparison are shown in figure 3. Based on the results, the mean visual analog pain scale at zero time (the burn) in silymarin and placebo groups were 9.8±1.2 and 9.5±2.2, respectively. The two groups were similar in this regard (P=0.14). The mean visual analog pain scale had no significant difference in any of the follow-up stages between the two groups (week 1: P=0.112, Week 2: P=0.43, Week 3: P=0.124, and week 4: P=0.44). However, the changes in the mean visual analog pain scale in both silymarin (P=0.001) and placebo (P=0.05) groups during 4 weeks of treatment significantly decreased (figure 5). Based on our results, no drug side effects were reported in the consumers of oral silymarin in the intervention group during the 4 weeks of treatment.

## Discussion

According to the present study, the basic treatment with oral silymarin for 4 weeks can be effective in healing the second-degree burning wounds. Although, adjuvant treatment with silymarin leads to a faster full recovery in patients with a second-degree burn. Therefore, our study showed cases of complete remission at the end of weeks 1, 2, 3 and 4 in silymarin group was significantly higher than the placebo group. Also, the patients’ pain during the 4 weeks of treatment and follow-up in both groups significantly decreased, and no difference was found between the two groups.

Jahromy et al. in 2011 conducted a study to evaluate the effect of silymarin on colon ulcer induced by acetic acid in mice. 32 adult mice in four groups have evaluated the effect of oral administration of silymarin 40 and 80 mg on colon ulcer. The samples of intervention group received daily silymarin 14 days before inducing the disease and then for a week after the disease. To induce colitis, 1 ml of 4% acetic acid was injected into the rectum. A week after the diagnosis of ulcers, a piece of the colon was removed for tissue examination and colonic injuries using Moretti’s method. Alkaline phosphatase levels were measured by ELISA method. 

The amount of tissue edema was also evaluated. The results showed that the concentration of alkaline phosphatase in the ulcer group was significantly higher than the control group and the silymarin group had significantly better-wound healing and less tissue edema (12) The results of this study are consistent with the present study in terms of wound healing. Sharifi et al. in 2012 conducted a study on the effect of silymarin as a local wound healing in a mouse model. In this study, excisional wounds were created in the skin on the back of the mice and divided the samples into 3 groups: control (no treatment), polyethylene glycol and the treatment group (receiving silymarin dissolved in polyethylene glycol) and compared them in days 5, 10 and 15 after starting the study. The wounds were evaluated in terms of histopathology and hydroxyproline level. The results showed that silymarin enhances epithelialization and reduces inflammation (10). Their results corresponded to ours in terms of healing of wounds.

Although few animal studies have been conducted on the effect of silymarin on wound healing (10, 11, 13), still, there are few studies on the effect of silymarin improving burn wound. Toklu et al. in 2007 performed a study on silymarin effect on oxidative damage caused by burns in rats. The results of this study showed that the burns caused increased levels of TNF-alpha and blood LDH. The MDA and GSH levels in skin sample 48 hours after the burns were respectively increased and decreased. Based on their results, both systemic and local silymarin can significantly reverse the high oxidant and antioxidant parameters (5). As well local silymarin significantly decreases the increased level of MPO activity and CL. Based on the conclusions of this study, local and systemic silymarin improves oxidative damage caused by burns in a mouse model, as a consequence silymarin can be considered as an adjunctive therapy or replacement in the burn wounds (14). 

Although our study was different in terms of methodology from the mentioned studies, their results were consistent with our study in terms of wound healing. Yet based on previous studies (15-17) and the present study on the impact of silymarin on wound healing, silymarin can be effective in improving various wounds and this drug may be used as adjunctive therapy in wound healing, but due to the lack of human studies, it is necessary to conduct more future studies in on different wounds to obtain comprehensive conclusion.

The limitations of this study included; the lack of examination of silymarin effect on oxidative factors; the deficiency of comparing the effect of local and oral silymarin and the unavailability of examination of silymarin effect on the infections in burn wound. As a result, future studies are recommended to consider these factors to obtain more functional conclusions.

In conclusion based on our findings, standard treatment and basic treatment along with oral silymarin for 4 weeks can be effective in healing the second-degree burns. Nonetheless, adjuvant treatment with silymarin leads to a faster complete remission in patients with second-degree burn surface. Silymarin can be considered as adjunctive therapy for burns’ wound healing.
